# Correlation between Exposure to UFP and ACE/ACE2 Pathway: Looking for Possible Involvement in COVID-19 Pandemic

**DOI:** 10.3390/toxics12080560

**Published:** 2024-07-31

**Authors:** Laura Botto, Alessandra Bulbarelli, Elena Lonati, Emanuela Cazzaniga, Paola Palestini

**Affiliations:** 1 School of Medicine and Surgery, University of Milano-Bicocca, 20900 Monza, Italy; laura.botto@unimib.it (L.B.); alessandra.bulbarelli@unimib.it (A.B.); elena.lonati1@unimib.it (E.L.); emanuela.cazzaniga@unimib.it (E.C.); 2 POLARIS Research Centre, University of Milano-Bicocca, 20900 Monza, Italy

**Keywords:** ultrafine particles (UFP), diesel exhaust particles (DEP), biomass combustion-derived particles (BB), inflammation, ACE2, COVID-19

## Abstract

The overlap between the geographic distribution of COVID-19 outbreaks and pollution levels confirmed a correlation between exposure to atmospheric particulate matter (PM) and the SARS-CoV-2 pandemic. The RAS system is essential in the pathogenesis of inflammatory diseases caused by pollution: the ACE/AngII/AT1 axis activates a pro-inflammatory pathway, which is counteracted by the ACE2/Ang(1-7)/MAS axis, which activates an anti-inflammatory and protective pathway. However, ACE2 is also known to act as a receptor through which SARS-CoV-2 enters host cells to replicate. Furthermore, in vivo systems have demonstrated that exposure to PM increases ACE2 expression. In this study, the effects of acute and sub-acute exposure to ultrafine particles (UFP), originating from different anthropogenic sources (DEP and BB), on the levels of ACE2, ACE, COX-2, HO-1, and iNOS in the lungs and other organs implicated in the pathogenesis of COVID-19 were analyzed in the in vivo BALB/c male mice model. Exposure to UFP alters the levels of ACE2 and/or ACE in all examined organs, and exposure to sub-acute DEP also results in the release of s-ACE2. Furthermore, as evidenced in this and our previous works, COX-2, HO-1, and iNOS levels also demonstrated organ-specific alterations. These proteins play a pivotal role in the UFP-induced inflammatory and oxidative stress responses, and their dysregulation is linked to the development of severe symptoms in individuals infected with SARS-CoV-2, suggesting a heightened vulnerability or a more severe clinical course of the disease. UFP and SARS-CoV-2 share common pathways; therefore, in a “risk stratification” concept, daily exposure to air pollution may significantly increase the likelihood of developing a severe form of COVID-19, explaining, at least in part, the greater lethality of the virus observed in highly polluted areas.

## 1. Introduction

On 11 March 2020, the World Health Organization (WHO) declared the coronavirus pandemic: SARS-CoV-2 (Severe Acute Respiratory Syndrome Corona Virus 2), the pathogenic agent of COVID-19, is now a health risk to the world’s population. Some early epidemiological studies have shown a positive correlation between air pollution and the spread of the virus [1]. 

The WHO has indicated that air pollution is responsible for widespread severe environmental and human health risks, and health problems can occur as a result of both short- and long-term exposures to these various pollutants [2]. 

Air pollution is the alteration of the internal or external environment by chemical, physical, or biological agents that modify the natural characteristics of the atmosphere. The pollutants with the strongest evidence for public health include particulate matter (PM), carbon monoxide (CO), ozone (O_3_), nitrogen dioxide (NO_2_), and sulfur dioxide (SO_2_). 

Particulate matter (PM), as a main component of air pollutants, contains a complex mixture of smoke, dust, and inhalable particles with different sizes, shapes, and chemical compositions. Conventionally, it is classified into two main classes based on their aerodynamic diameter (d*a*): PM10 (da < 10 µm) and PM2.5 (da < 2.5 µm) [3,4].

In 2020, Wu and coauthors [5] proved that long-term exposure to PM2.5 was associated with a 15% increase in the mortality rate from COVID-19 after accounting for many area-level confounders. Moreover, long-term exposure to NO_2_ and short- and long-term exposure to PM2.5 appear to be most consistently associated with COVID-19 epidemiological and clinical data worldwide, according to a recent systematic retrospective review [6].

PM2.5 predominates in winter. It is mainly produced by combustion processes and is, therefore, enriched in polycyclic aromatic hydrocarbons (PAHs) [7,8,9,10]. This fraction is more harmful than larger ones because it is more efficiently retained in the alveolar lungs portion and contains a significant amount of ultrafine particles, UFP (d*a* < 0.1 um). 

The main mediators of PM toxicity are UFPs, as they have a better efficacy of penetration into the respiratory system and a greater capacity for translocation from the airways to the blood circulation [11,12]. 

In Lombardy (Northern Italy), the main sources of PM2.5 are the combustion of solid biomass for domestic heating (49.8%) and the combustion of diesel for private and public transport (21.5%) [13,14]. These processes are considered to be significant contributors to UFP emissions. They mainly produce particles 15–30 nm in diameter, which are often aggregated [15,16,17]. 

In two recent publications [18,19], we have shown that the chemical composition of UFP affects the activation of different responses mediated by their components or by the production of pro-inflammatory and pro-oxidative molecules in the brain and respiratory and cardiovascular systems.

This study of the role of air pollution in COVID-19 has led to the postulation of three main hypotheses [20]. 

In the first hypothesis, COVID-19 could be airborne like SARS-CoV-1. It would be transported over greater distances than those considered for close contact [21]. PM could create an environment suitable for transporting the virus in the most polluted areas. 

On the other hand, exposure to PM is known to cause oxidative stress by stimulating ROS production, which leads to cell damage and induces inflammatory mechanisms following activation of the immune response [22,23]. In the lungs, in particular, pollutants alter the immune system by modulating the antiviral response of alveolar macrophages. Recent studies have shown that exposure to pollutants reduces the phagocytic capacity of macrophages and, thus, their ability to properly inactivate viruses [24,25]. Based on the second hypothesis, this cellular state could increase the susceptibility and severity of symptoms in COVID-19 patients as the new coronavirus unleashes an inflammatory storm [26] that is more fueled in the case of pre-exposure to pollutants.

A third hypothesis has been postulated since 2018 when Lin and coauthors [27] showed that PM2.5 exposure induced a significant increase in ACE2 (angiotensin-converting enzyme 2) in the lungs of mice; however, it also acts as a receptor for SARS-CoV-2 endocytosis [28]. ACE2 is a membrane enzyme found in the lungs, arteries, heart, kidney, and intestine cells that converts angiotensin 2 peptide to angiotensin 1-7, Ang(1-7), a vasodilatory and anti-inflammatory molecule, that regulates blood pressure [29]. ACE2 and ACE are important components of the renin–angiotensin system (RAS), which is an endocrine system that regulates cardiovascular physiology and blood pressure and plays a fundamental role in repairing the inflammatory and damage processes.

Thus, ACE2 overexpression after PM exposure may potentially increase the risk of COVID-19 infection, where ACE2 is the door to the virus’s entry [30]. Moreover, by binding to ACE2, the virus blocks its activity, which is crucial for immune defense and protection against inflammation, the main cause of death in COVID-19.

To date, the three hypotheses, which are not mutually exclusive, are still “open”, and few in vitro and/or in vivo studies have addressed the relationship between air pollution and COVID-19 from a molecular point of view [31,32,33]. 

Therefore, in a recent publication, we evaluated in an in vivo model whether RAS could be the *trait d’union* between air pollution and COVID-19 by assessing the ACE2 and ACE expression levels, as well as those of inflammatory markers (cyclooxygenase 2, COX-2) and oxidative stress markers (heme oxygenase-1, HO-1, and inducible nitric oxide synthase, iNOS), following exposure to PM2.5 [34]. 

The analysis was performed in the lungs and other organs involved in the COVID-19 syndrome. Indeed, we have demonstrated that sub-acute exposure to PM2.5 altered the ACE/ACE2 system, with possible consequences for COVID-19 pathogenesis. The UFP, a significant component of PM2.5, has a greater penetration and translocation due to its size of less than 100 nm, which could possibly induce a worse toxicity profile at the systemic level. For this reason, it would be interesting to repeat the same analyses already carried out with PM2.5. In this work, an in vivo BALB/c male mouse model was used to analyze the effects of exposure to UFP derived from different anthropogenic sources (biomass burning-derived particles, BB and diesel exhaust particles, DEP), administered by intratracheal instillation. Single exposures (acute treatment) were performed to reveal the short-term effects of UFP, mostly related to oxidative stress and inflammation, while three repeated instillations (sub-acute treatment) were performed because it is known [9,18,19,35,36,37] that marker changes at the systemic level are possibly due to ultrafine components and/or mediators translocation from the first site of deposition and are considered representative of subacute exposure [9].

## 2. Materials and Methods

### 2.1. Materials

All commercial chemicals were of the highest available grade and were acquired from Sigma Chemical Co. (Milano, Italy). All solutions for cell culture were from Euroclone (Celbio, Milano, Italy). Precision Plus Protein Standards (All Blue) were from Bio-Rad (Milano, Italy). The complete protease inhibitor cocktail was from Roche Diagnostics S.p.A (Milano, Italy). The nitrocellulose membrane was from Amersham (GE Healthcare Europe GmbH, Milano, Italy).

Primary antibodies anti-ACE2 and anti-ACE were from R&D Systems (Minneapolis, MN, USA); anti-COX2 was from BD Transduction Laboratories (Newark, NJ, USA); anti-HO-1 was from Cell Signaling Technology (Danvers, MA, USA), and anti-iNOS was from Byorbyt (Cambridge, UK). Secondary antibodies rabbit anti-goat, goat anti-mouse or anti-rabbit (HRP)-conjugated, and ECL SuperSignal detection kit were from Thermofisher Scientific (Milano, Italy). 

### 2.2. Animal Housing

Male BALB/c OlaHsd mice (7–8 weeks old, 20–25 g weight) were purchased from Envigo (San Pietro al Natisone, Udine, Italy) and housed in groups of three in plastic cages for five days. The housing facility was under controlled environmental conditions (temperature 19–21 °C, humidity 40–70%, lights on 7 a.m.–7 p.m.), and food and water were administered ad libitum. The Institutional Animal Care and Use Committee of the University of Milano-Bicocca approved the protocol and procedures (protocol 02-2014) that complied with guidelines set by the Italian Ministry of Health (DL 26/2014 “Application of the Directive n. 2010/63/EU on the protection of animals used for a scientific purpose”).

### 2.3. UFP Characterization

DEP and BB batches were provided by ENEA (Agenzia Nazionale per le Nuove Tecnologie, l’Energia e lo Sviluppo Economico Sostenibile) in the framework of the project “Biological effects and human health impacts of ultrafine particle sources” lead by POLARIS research center. Particle sampling procedures and details of the complete UFP composition have already been published [17]. 

DEPs were sampled from a Euro 4 light-duty vehicle without DPF run over a chassis dyno according to the “URBAN” Artemis Driving Cycle, which represented real average stop-and-go driving conditions typical of a European city urban context. Several URBAN cycles were performed with thoroughness to collect a suitable mass of particles for biological and chemical analyses. UFPs were collected on Teflon filters (Whatman pure Teflon filters) using a DGI-1570 (Dekati Gravimetric Impactor, Finland) to remove bigger aggregates. Teflon filters were inserted in the last stage of the impactor and secured by custom design support.

BB particulate emissions from a modern automatic 25 kW pellet boiler were sampled on Teflon filters after dilution of flue gases with clean air to improve volatile organic compound condensation.

The dilution rate was evaluated a posteriori, considering the CO_2_ content in the flue gases and the sampling gases. The levels of gaseous emissions were characterized using a multiparametric gas analyzer, Horiba PG250 (Horiba, Japan). After sampling, DEP and BB filters were kept at −20 °C immediately until chemical characterization or UFP extraction for biological tests.

UFP morphology was characterized by transmission electron microscopy (TEM) and scanning electron microscopy (SEM). Particles were transferred from sampling filters to Formvar^®^ coated 200-mesh copper grids by gently rubbing grids on the filter surfaces. The grids were directly inserted into a Jeol JEM-1220 TEM (Boston, MA, USA) equipped with a CCD camera for TEM analysis, or they were mounted onto stubs for SEM (LEO 1430) investigations. Energy-dispersive X-ray analysis (EDX) coupled to SEM was used to investigate the elemental composition of particles. The accuracy level of the Oxford EDX probe was 1%.

In both BB and DEP samples, aggregates of round carbonaceous particles lower than 50 nm in diameter were completely dissolved in aqueous media. 

PAHs and transition metal (Fe, Zn, Cr, Pb, V, and Ni) concentrations were high in DEP compared to BB. A detailed composition is summarized in Table S1 (Supplementary Materials).

### 2.4. Intratracheal Instillation

Animals were randomly divided into three experimental groups (three mice for each group) and exposed, in the morning, to acute and sub-acute treatments with isotonic solution (sham), BB-particles (BB-treated mice), and DEP particles (DEP-treated mice). The experiments were replicated twice, for a total of 6 shams, 6 BB-, and 6 DEP-treated mice for each type of treatment. The sample size was chosen to minimize the number of animals employed, according to the 3R principle [38]. Every mouse was singularly exposed to a mixture of 2.5% isoflurane (flurane) anesthetic gas and kept under anesthesia during the whole instillation procedure. Furthermore, the solution containing the UFP suspended in isotonic saline solution was stirred on a vortex and sonicated on ice by immersion in a Branson 2510 ultrasonic sonicator for 2 min at moderate energy. Everything was repeated 2 times, immediately before the instillation [39]. Intratracheal instillations with 50 µg of BB or DEP in 100 µL of isotonic saline solution or with 100 µL of isotonic saline solution were achieved using a MicroSprayer^®^ Aerosolizer system (MicroSprayer^®^ Aerosolizer- Model IA-1C and FMJ-250 High-Pressure Syringe, Penn Century, USA; validated by Bivas-Benita and coauthors [40], as previously described [18,19,36,37,41,42]. The dose of 50 µg of BB or DEP in 100 µL of isotonic saline solution was selected as the best dose to test the effects of acute and sub-acute UFP treatment, as suggested by Kaewamatawong and coauthors [43]. The authors suggested a 50 ug/mice dose as the most appropriate for detecting acute and sub-acute inflammatory changes in lung exposition. The UFP dose used here is the lowest dose to establish lung inflammatory response in exposed mice. This dose is the same as that of our previous works [18,19], similarly reported in the literature [43,44]. 

Immediately following instillation, treated and sham mice were allowed to recover under visual control and kept under controlled environmental conditions.

**Figure 1:**
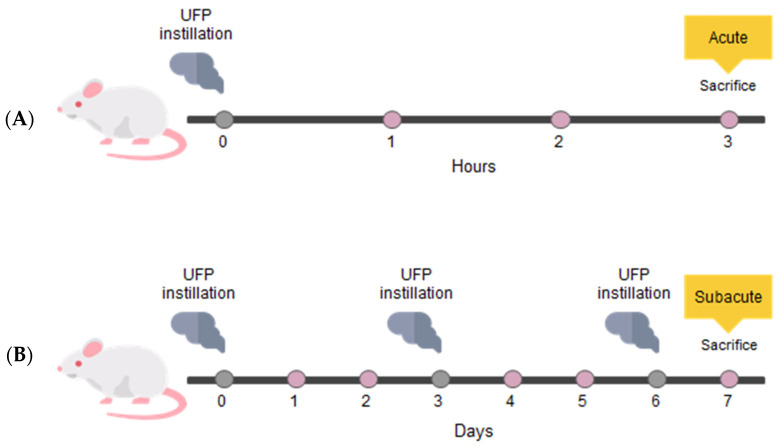
Study design. Schematic representation of BALB/c acute (**A**) and sub-acute (**B**) treatments. (modified by Massimino et al. [46]).

Acute and sub-acute treatment protocols are illustrated in Figure 1, as previously described [18]. The acute treatment consisted of a single instillation and animal sacrifice after 3 h. Instead, the sub-acute treatment consisted of three repeated instillations on days 0, 3, and 6 and animal sacrifice 24 h following the last instillation [9,34,45]. Mice of each experimental group were anesthetized by gas to minimize suffering and euthanized with cervical dislocation.

### 2.5. Organ Homogenization

All the procedures were performed on ice. The lungs, heart, liver, kidneys, and brain from sham and UFP-treated mice were washed in ice-cold isotonic saline solution immediately after being excised. Organs were minced, suspended in 0.9% NaCl plus protease inhibitors cocktail (Complete, Roche Diagnostics S.p.A, Milano, Italy), homogenized for 30 s at 11,000 rpm with Ultra-Turrax T25 basic (IKA WERKE, Staufen im, Breisgau, Germany), and sonicated for 30 s. The samples were stored at −20 °C.

### 2.6. Electrophoresis and Immunoblotting

The homogenates obtained from all the organs of sham and UFP-treated mice were analyzed for protein content by quantification with a micro-bicinchoninic acid (BCA) assay (Sigma-Aldrich Cat# B9643, Cat# C2284, St. Louis, MO, USA); then, 30 µg of total proteins for each sample was subjected to SDS-PAGE (10%). Proteins were transferred by Western blot to a nitrocellulose membrane and revealed by Ponceau staining to evaluate proper transfer. Immunoblottings were performed using specific antibodies: ACE2 (2.5 µg/mL); ACE (0.05 µg/mL); COX-2 (1:250); HO-1 (1:1000); iNOS (1:300). The secondary antibodies were appropriate horseradish peroxidase (HRP)-conjugated rabbit anti-goat (1:4000) and goat anti-rabbit or anti-mouse (1:5000). Immunoreactive proteins were revealed by enhanced chemiluminescence (ECL) and semi-quantitatively estimated by ImageQuant™ 800 (GE Healthcare Life Sciences, Milan, Italy), program 1D gel analysis. No blinding was performed. Staining of total proteins versus a housekeeping protein represents the actual amount of loading more accurately due to minor procedural and biological variations, as demonstrated by recent studies [47,48]. Accordingly, samples were normalized with respect to the total amount of proteins detected by Ponceau staining, allowing for a straightforward correction for lane-to-lane variation [48,49]. Each protein was then expressed as a percentage of the sham, which represented the control.

### 2.7. Blood Analysis

Blood of sham and UFP-treated mice was collected using intracardiac puncture with the anticoagulant (EDTA, Na-citrate). Plasma was obtained after two centrifugations: the first at 2000× *g* for 20 min and the second at 10,000× *g* for 10 min at 4 C to altogether remove platelets. Plasma samples have been then submitted to immunoblot assay of s-ACE2 (1:200, Santa Cruz Biotechnology, Dallas, TX, USA) according to previously described protocols. All the data were normalized concerning the total amount of proteins detected by Ponceau staining and expressed as a percentage of the sham, representing the control.

### 2.8. Statistical Analysis

The means (±standard error of the mean, S.E.) were calculated for each parameter measured in sham and UFP-treated mice. Statistical differences were tested by one-way ANOVA and a *t*-test and were considered significant at the 95% level (*p*-value < 0.05).

## 3. Results

Following the analysis of the impact of sub-acute exposure to PM2.5 in the ACE/ACE2 system [34], we extended this study to the effects of acute and sub-acute exposure to UFP. 

In this study, male BALB/c mice were instilled intratracheally with UFP deriving from different anthropogenic sources (BB and DEP). The consequences of acute and sub-acute exposure on the ACE/ACE2 system were assessed in the lungs and other organs involved in the COVID-19 syndrome, such as the heart, liver, kidney, cerebellum, and cerebral cortex. The results of this study are particularly relevant as they provide insights into the extra-pulmonary effects of SARS-CoV-2 infection, a key aspect of COVID-19 syndrome.

Furthermore, the effects of UFP exposure on COX-2, HO-1, and iNOS levels were analyzed in the liver and kidney to complement and support previous works in which UFP-exposed mice showed an increase in COX-2, HO-1, and iNOS in the lungs and heart [18], as well as in the cerebellum and hippocampus [19].

### 3.1. Lungs

**Figure 2:**
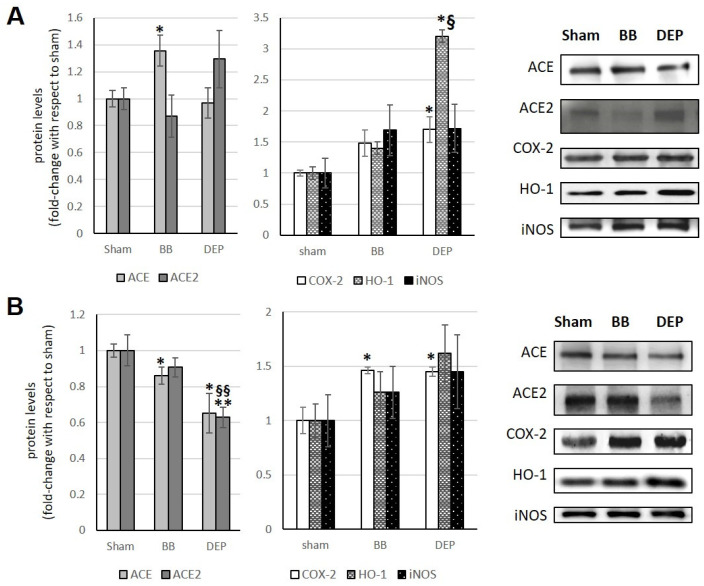
Immunoblotting analysis of ACE, ACE2, COX-2, HO-1, and iNOS (modified from Farina et al. [18]) in the lungs. Proteins were evaluated following cell acute (**A**) or sub-acute (**B**) treatment with UFP (BB or DEP) and have been normalized to the total amount of proteins detected by Ponceau staining, allowing for a straightforward correction for lane-to-lane variation. Histograms display each protein as fold-change to sham with the corresponding representative immunoblotting images. Data are expressed as mean ± s.e. standard error (n = 6).; * *p* <0.05 versus sham, ** *p* < 0.01 versus sham; § *p* < 0.05 versus BB; §§ *p* < 0.01 versus BB.

Following acute exposure to DEP, an increase in ACE2 is observed compared to sham, although not significant. Furthermore, acute exposure to BB determines a significant increase in ACE (+35.6%) (Figure 2A). Instead, the sub-acute treatment with both UFPs induces a decrease in ACE (−14% and −34.9% with BB and DEP, respectively), which after DEP exposure is accompanied by a simultaneous decrease in ACE2 (−37.1%), also compared to BB exposure (−27.8%), without significant changes in the ACE/ACE2 ratio (Figure 2B; Table 1). 

Previous studies [18] showed that COX-2 increased by 46% compared to the sham after BB exposure only in sub-acute treatment, whereas the protein always increased after DEP exposure in both acute and sub-acute treatments (+70% and +45%, respectively). In addition, acute DEP administration caused a relevant increase in HO-1 compared to both sham and BB treatments (+220% and +180%, respectively). Conversely, iNOS did not change after any treatment (Figure 2A,B).

### 3.2. Heart

**Figure 3:**
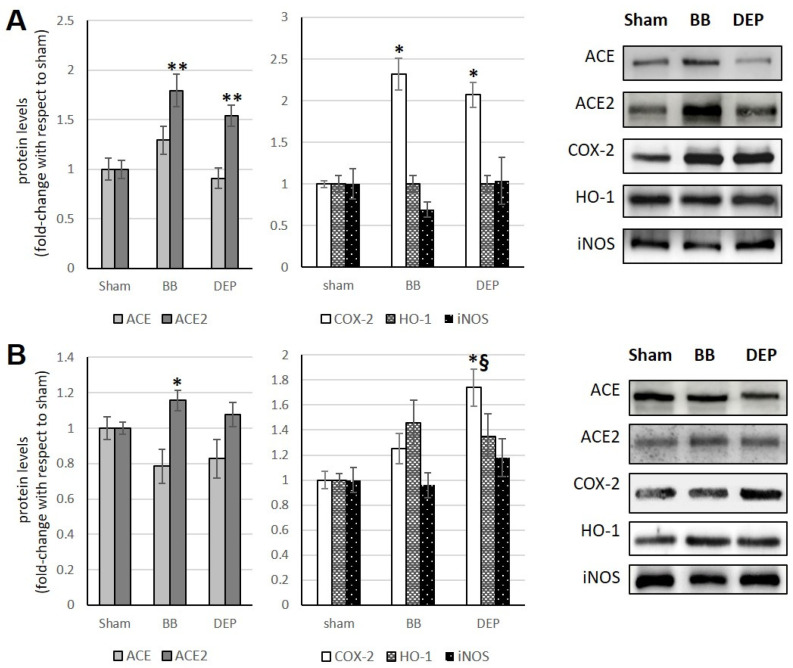
Immunoblotting analysis of ACE, ACE2, COX-2, HO-1, and iNOS (modified from Farina et al. [18]) in the heart. Proteins were evaluated following cell acute (**A**) or sub-acute (**B**) treatment with UFP (BB or DEP) and have been normalized to the total amount of proteins detected by Ponceau staining, allowing for a straightforward correction for lane-to-lane variation. Histograms display each protein as fold-change to sham with the corresponding representative immunoblotting images. Data are expressed as mean ± s.e. standard error (n = 6); * *p* < 0.05 versus sham; ** *p* < 0.01 versus sham; § *p* < 0.05 versus BB.

Acute and sub-acute exposure to BB causes an increase in ACE2 (+79.4% and +15.7%, respectively) in treated mice compared to sham, whereas DEP treatment causes a significant rise in ACE2 (+53.8%) only in acute exposure (although a slight increase is also observed in sub-acute exposure) (Figure 3A,B). The increase in ACE (+29.3%) in acute BB exposure, although not significant, counterbalances the increase in ACE2, leaving the ACE/ACE2 ratio unchanged. Instead, a significant decrease in the ACE/ACE2 ratio is observed after DEP treatments (Figure 3A,B; Table 1). 

Studies by Farina and coauthors [18] showed that, unlike the lungs, the heart showed a significant increase in COX-2 (+132%) after acute exposure to BB compared to sham. Instead, as in the lungs, COX-2 always increased in the heart after DEP treatment (+107% and +74% in acute and sub-acute, respectively), compared to sham and, in particular, in the sub-acute treatment also compared to BB exposure (+49%). Furthermore, UFP never induced an increase in HO-1 and iNOS in treated mice compared to sham (Figure 3A,B).

### 3.3. Liver

**Figure 4:**
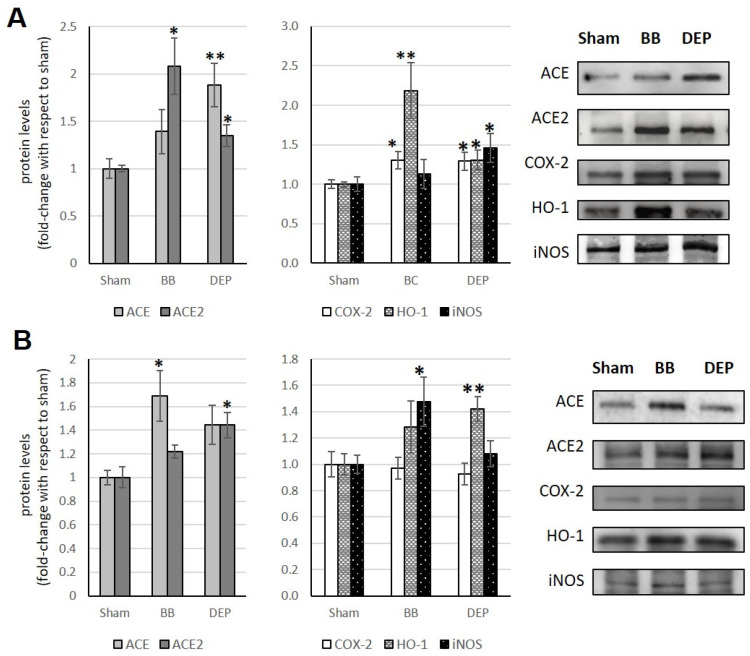
Immunoblotting analysis of ACE, ACE2, COX-2, HO-1, and iNOS in the liver. Proteins were evaluated following cell acute (**A**) or sub-acute (**B**) treatment with UFP (BB or DEP) and have been normalized to the total amount of proteins detected by Ponceau staining, allowing for a straightforward correction for lane-to-lane variation. Histograms display each protein as fold-change to sham with the corresponding representative immunoblotting images. Data are expressed as mean ± s.e. standard error (n = 6); * *p* < 0.05 versus sham; ** *p* < 0.01 versus sham.

Exposure to UFP induces ACE2 changes in the liver, which appears to be the opposite of what occurs in the heart. Indeed, BB treatment results in an ACE2 increase (+108%) only in acute exposure, while DEP exposure consistently leads to significant increases in ACE2 levels (+34.4% in acute and +44.2% in sub-acute vs. sham) (Figure 4A,B). Additionally, DEP acute exposure appears to influence the ACE/ACE2 ratio in the liver, as observed in Table 1. 

Acute exposure to BB and DEP induces a significant COX-2 increase, by about 30% in both cases, in treated mice compared to sham. Furthermore, BB acute exposure causes a significant HO-1 increase (+118.7%), while DEP always causes the HO-1 increase, both in acute and sub-acute treatments (+30.7% and +42.1%, respectively). Finally, sub-acute treatment with BB and acute treatment with DEP show, in both cases, an increase in iNOS, (about +50%) Figure 4A,B.

### 3.4. Kidneys

**Figure 5:**
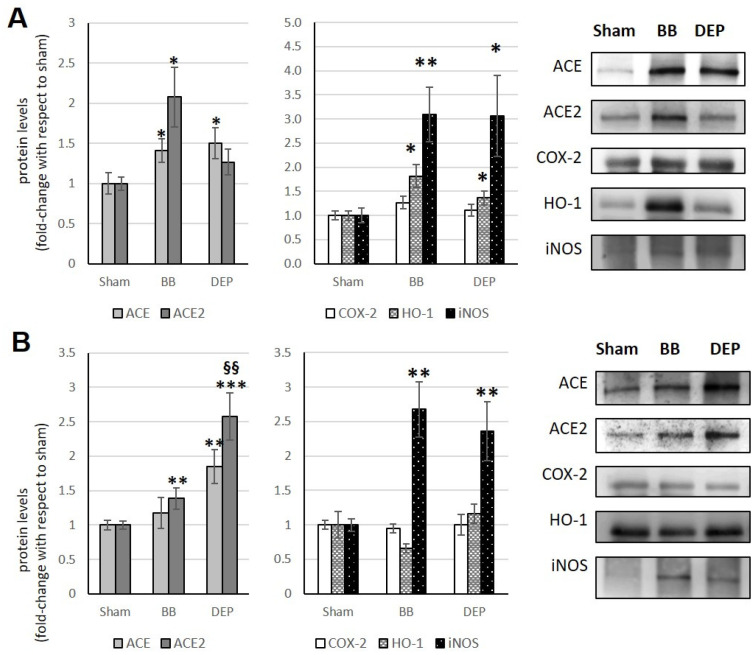
Immunoblotting analysis of ACE, ACE2, COX-2, HO-1, and iNOS in the kidneys. Proteins were evaluated following cell acute (**A**) or sub-acute (**B**) treatment with UFP (BB or DEP) and have been normalized to the total amount of proteins detected by Ponceau staining, allowing for a straightforward correction for lane-to-lane variation. Histograms display each protein as fold-change to sham with the corresponding representative immunoblotting images. Data are expressed as mean ± s.e. standard error (n = 6); * *p* < 0.05 versus sham; ** *p* < 0.01 versus sham; *** *p* < 0.001 versus sham; §§ *p* < 0.01 versus BB.

In the kidneys, following BB acute exposure, there is a significant increase in both ACE2 (+107.7%) and ACE (+40.9%) levels compared to the sham group. DEP, in acute exposure, results in a significant increase only in ACE level (+50.6%) (Figure 5A). 

Conversely, in the sub-acute treatment, BB leads to a significant ACE2 increase (+38.6%), while DEP induces a significant rise in both ACE2 (+157.4% and +118.8% compared to sham and BB treatment, respectively) and ACE (+84.7% compared to sham) (Figure 5B). 

Table 1 shows that at the renal level, exposure to the different UFPs does not result in any alterations to the ACE/ACE2 ratio. 

Furthermore, acute treatment with both UFPs has been observed to cause an increase in HO-1 (+81.3% and +36.1% with BB and DEP, respectively) and iNOS levels (about +210% with both UFPs). On the other hand, sub-acute treatment with both UFPs induces only an iNOS increase (+167.4% and +135.9% with BB and DEP, respectively) (Figure 5A,B).

### 3.5. Cerebral Cortex

**Figure 6:**
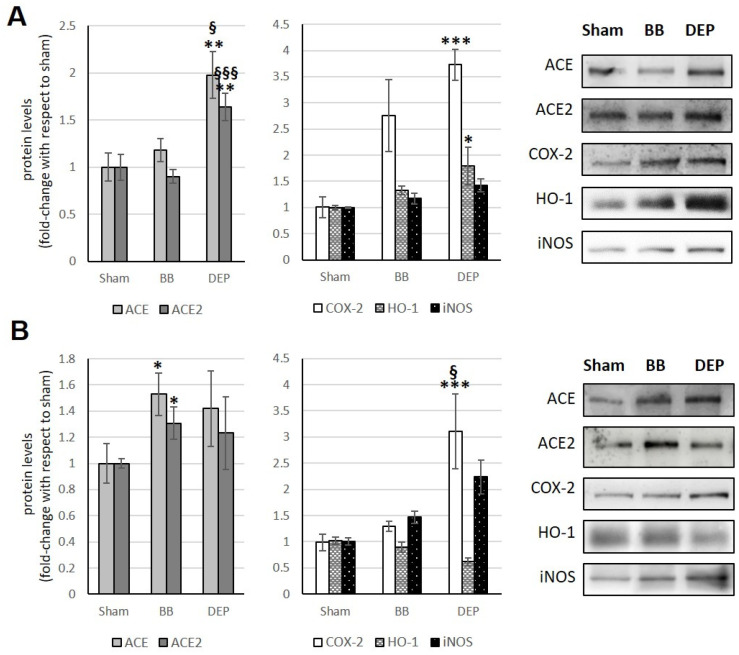
Immunoblotting analysis of ACE, ACE2, COX-2, HO-1, and iNOS (modified from Milani et al. [19]) in the cerebral cortex. Proteins were evaluated following cell acute (**A**) or sub-acute (**B**) treatment with UFP (BB or DEP) and have been normalized to the total amount of proteins detected by Ponceau staining, allowing for a straightforward correction for lane-to-lane variation. Histograms display each protein as fold-change to sham with the corresponding representative immunoblotting images. Data are expressed as mean ± s.e. standard error (n = 6); * *p* < 0.05 versus sham; ** *p* < 0.01 versus sham; *** *p* < 0.001 versus sham; § *p* < 0.05 versus BB; §§§ *p* < 0.001 versus BB.

In the cerebral cortex, BB acute exposure does not alter ACE and ACE2 levels. DEP, on the other hand, leads to a significant increase in both ACE2 (+63.9% and +73.9% compared to sham and BB treatment, respectively) and ACE (+97.7% and +79.9% compared to sham and BB treatment, respectively). This results in a consequent ACE/ACE2 ratio maintenance. Conversely, BB sub-acute exposure causes a significant ACE and ACE2 increase (+52.9% and +30.7%, respectively) with a consequent ACE/ACE2 ratio maintenance. In comparison, DEP has been demonstrated to exert no influence on the levels of either (Figure 6A,B; Table 1).

Furthermore, as demonstrated by Milani and coauthors [19], BB exposure did not markedly alter COX-2, HO-1, and iNOS levels. Instead, following both acute and sub-acute DEP treatments, a marked elevation in COX-2 levels was observed in comparison to the sham group (+272.9% and +210.4%, respectively), particularly in the sub-acute group in relation to BB (+181%). Additionally, acute DEP exposure resulted in a significant increase in HO-1 levels (+79.6%) in treated mice compared to the sham group, whereas no significant changes were observed in iNOS levels (Figure 6A,B).

### 3.6. Cerebellum

**Figure 7:**
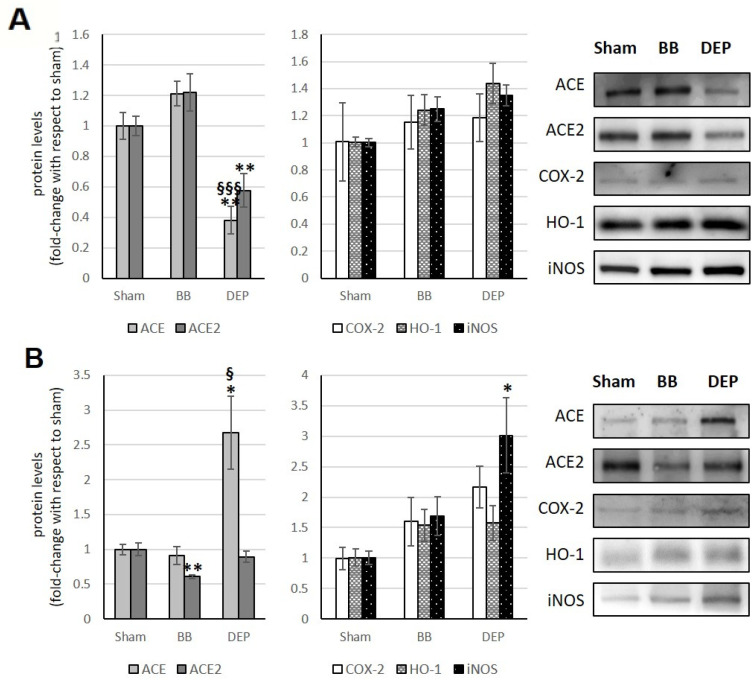
Immunoblotting analysis of ACE, ACE2, COX-2, HO-1, and iNOS (modified from Milani et al. [19] in the cerebellum. Proteins were evaluated following cell acute (**A**) or sub-acute (**B**) treatment with UFP (BB or DEP) and have been normalized to the total amount of proteins detected by Ponceau staining, allowing for a straightforward correction for lane-to-lane variation. Histograms display each protein as fold-change to sham with the corresponding representative immunoblotting images. Data are expressed as mean ± s.e. standard error (n = 6); * *p* < 0.05 versus sham; ** *p* < 0.01 versus sham; § *p* < 0.05 versus BB; §§§ *p* < 0.001 versus BB.

Following acute exposure, DEP results in a significant reduction in both ACE (−62% and −83% compared to sham and BB treatment, respectively) and ACE2 (−42.4%). These changes do not alter the ACE/ACE2 ratio (Figure 7A and Table 1). Similarly, the significant decrease in ACE2 (−39.3%) after sub-acute exposure to BB does not modify the ACE/ACE2 ratio, as it is accompanied by a decrease in ACE, although not statistically significant.

Conversely, sub-acute DEP exposure leads to an increase in the ACE/ACE2 ratio due to a significant increase in ACE (+167.9% and +176.5% compared to both sham and BB treatments, respectively) (Figure 7B and Table 1).

### 3.7. Plasma

**Figure 8:**
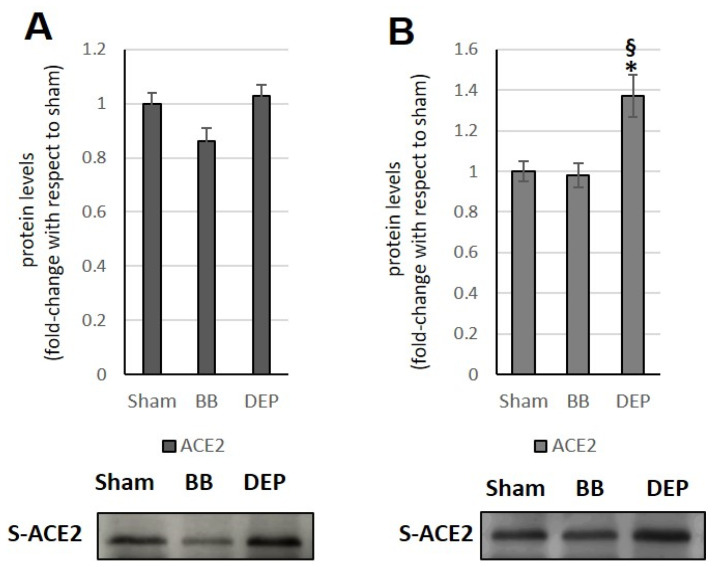
Immunoblotting analysis of s-ACE2 in the plasma. Protein was evaluated following cell acute (**A**) or sub-acute (**B**) treatment with UFP (BB or DEP) and has been normalized to the total amount of proteins detected by Ponceau staining, allowing for a straightforward correction for lane-to-lane variation. Histograms display each protein as fold-change to sham with the corresponding representative immunoblotting images. Data are expressed as mean ± s.e. standard error (n = 6); * p < 0.05 versus sham; § *p* < 0.05 versus BB.

Finally, the analyses performed on the plasma of animals following sub-acute DEP exposure showed a significant increase (approximately +40% compared to both sham and BB treatments) in soluble ACE2 (s-ACE2) (Figure 8A,B).

## 4. Discussion

Air pollution is known to affect almost every organ system, causing a wide range of effects, from asthma symptoms and exacerbation to illness and death from ischemic heart disease, lung cancer, COPD, lower respiratory infections, stroke, and type 2 diabetes [50]. Furthermore, epidemiologic evidence suggests that PM, especially PM2.5, increases the risk of COVID-19 infection, severity, and death [5,6]. 

Two recent papers, [51,52], have investigated the association between long-term exposure to air pollutants and mortality among 4 million COVID-19 cases in Italy from February 2020 to June 2021. The authors have estimated that ∼8% of COVID-19 deaths were attributable to pollutant levels above the WHO 2021 air quality guidelines [53]. The meta-analysis of Sheppard and coauthors [1], which included 18 studies, showed that a 10 μg/m^3^ increase in PM2.5 was associated with 66% of COVID-19 infections and 127% of severe illness.

These and other studies in different world areas have correlated air pollution levels with disease severity, but the reason is not well understood [1,54].

In this work, we have shown that the RAS is one of the molecular mechanisms involved in this correlation. The two key enzymes of the RAS, ACE2 and ACE, are expressed in many organs (lungs, heart, kidney, etc.) and generate two cellular pathways with opposite effects. In the ACE2 pathway (ACE2/Ang(1-7)/MASR), Ang(1-7) produced by ACE2 from AngI interacts with the MAS receptor, which, in turn, suppresses the transduction pathway of STAT3 and ERK. This reduces the expression of MMPs and pro-inflammatory molecules. Consequently, this pathway has an anti-inflammatory role [55]. Conversely, in the ACE pathway (ACE, AngII, AT1R), AngII produced by ACE from AngI interacts with the AT1 receptor and induces the expression of IL-6, TNF-α, and TGF-β1. These cytokines stimulate the activation of the STAT3 and ERK transduction pathways, increasing the production of MMPs and pro-inflammatory molecules. Thus, this pathway is pro-inflammatory [55]. 

On the other hand, ACE2 is the receptor through which SARS-CoV-2 enters host cells to replicate and form mature virions [56]. Following this interaction, the virus-ACE2 complex is internalized with consequent down-regulation of anti-inflammatory and organ-protective pathways [56]. In the lungs, DEP acute exposure induces a slight increase in ACE2, which, although not significant, could potentially facilitate SARS-CoV-2 entry in the event of infection. Furthermore, the COX-2 increases [18] indicate a strong inflammatory state, which could potentially be exacerbated by an underlying infection. The COX-2 up-regulation is a distinctive feature of viral infections, particularly in the context of acute respiratory syndrome induced by SARS-CoV-2, which can lead to severe tissue damage [57]. Concurrently, the HO-1 increase suggests a potential attempt at an oxidative stress reaction in the lungs [18]. 

Instead, a significant increase in ACE levels is observed following acute exposure to BB, which could indicate a compromised lung condition. This trend has been linked to acute respiratory distress syndrome (ARDS) in animal models [58].

The sub-acute exposure of both UFPs induces an ACE decrease and a COX-2 increase. This enzyme produces pro-inflammatory lipid mediators, which cause the cytokine storm that mediates the extensive inflammation and organ damage in patients with severe COVID-19 [57].

DEP significantly reduces ACE2 levels, suggesting a diminished anti-inflammatory and organ-protective effect. This could be a critical issue as it is known that the severity of COVID-19 is related to the increase in inflammatory mediators [26].

Several studies have demonstrated that the ACE2/Ang(1-7)/MASR axis plays a protective role in lung pathophysiological processes. The activation of this pathway provides a shield against the adverse effects mediated by RAS or by the ACE/AngII/AT1R axis, such as lung inflammation, fibrosis, pulmonary arterial hypertension, and alveolar epithelial cell apoptosis [59]. This underscores the potential anti-inflammatory and organ-protective role of ACE2. In the case of the heart, the increases in ACE2 levels observed after UFP exposure (except for sub-acute DEP exposure) could be interpreted as a protective response. Several studies have highlighted the cardioprotective effect of the ACE2/Ang(1-7)/MASR axis against damage by the ACE/AngII/AT1R axis. Ferreira and coauthors [60] showed that activation of the ACE2-mediated pathways leads to a significant reduction in cardiac arrhythmias induced by ischemia/reperfusion (anti-arrhythmogenic effect) and an improvement in post-ischemic cardiac functions. Subsequently, Souza-Santos and coauthors [59] highlighted the ability of Ang(1-7) to attenuate cardiac hypertrophy, indicating its anti-hypertrophic role.

However, the increase in ACE2 always suggests a greater vulnerability to SARS-CoV-2 entry in case of contact with the virus. Similarly, to what has been observed in the lungs, the level of COX-2 increases following DEP exposure, while only BB acute exposure causes a significant COX-2 increase [18]. 

The data appear to suggest that UFP may contribute to the development of an inflammatory condition at the cardiac level. In the case of SARS-CoV-2 infection, the elevated COX-2 levels may render the heart a “fragile” organ, which may contribute to the observed susceptibility in patients with severe COVID-19 versus moderate forms [61].

Treatment of the liver with UFP leads to a similar situation with the heart. Again, the increase in ACE2 observed after UFP exposure (except for sub-acute BB exposure) can be interpreted as a protective response. In parallel, DEP acute exposure and BB sub-acute exposure cause a rise in ACE. This trend causes an imbalance in the ACE/ACE2 ratio in favor of ACE2 in DEP-acute-treated mice.

Interestingly, sub-acute exposure to PM2.5 induced the same trends, leading to a significant increase in the ACE/ACE2 ratio and, as a consequence, in pro-inflammatory pathways. This finding suggests that this organ is very sensitive to air pollution.

Numerous studies document the beneficial effects of the ACE2/Ang (1-7)/MASR axis in the liver, such as improvements in steatosis and non-alcoholic inflammation, liver fibrosis, and insulin sensitivity [59]. These observations are consistent with the increase in ACE2 in chronic liver lesions in animal and human models. In addition, Ang(1-7) is known to suppress the growth of hepatocellular carcinoma and angiogenesis [59]. Lubel and coauthors [62] demonstrated that patients with liver cirrhosis had elevated plasmatic levels of AngII and Ang(1-7). In an in vivo model of liver cirrhosis, Ang(1-7) significantly inhibits the vasoconstriction induced by intrahepatic AngII through the NO signaling pathway dependent on eNOS and guanylate cyclase [63].

On the other hand, the increase in ACE2 after exposure to UFP could promote binding to the virus, and the parallel rise in an exacerbated inflammatory state, as also evidenced by the increase in iNOS, suggests a greater probability of organ damage in the event of SARS-CoV-2 infection.

In the liver of HCV patients, the higher expression of iNOS induces the excessive formation of NO and positively correlates with liver inflammation and viral load [64]. 

Many studies have shown that the expression of ACE2 in the kidney regulates hypertension and vasoconstriction induced by activation of the ACE/AngII/AT1R pathway and that activation of the ACE2/Ang(1-7)/MASR pathway promotes vasodilation [59]. Consequently, the increase in renal ACE2 levels (except for acute DEP exposure) may be interpreted as a protective response to UFP treatment, especially for the parallel increase in ACE (except for sub-acute BB treatment).

Acute exposure to both UFPs causes an increase in HO-1, which, apparently, does not counteract oxidative stress, as suggested by the concomitant increase in iNOS. A significant increase in iNOS was also observed after sub-acute exposure to both UFPs, demonstrating that the kidneys are particularly affected by pollutants.

Indeed, in a mouse model, iNOS and AngII are implicated in acute renal failure induced by LPS, and their down-regulation significantly improves the clinical picture [65]. Consequently, it might be the case that exposure to air pollution inducing the ACE2 and iNOS increase could impair kidney function over time and worsen following SARS-CoV-2 infection.

In the cerebral cortex, ACE2 levels increase after DEP acute and BB sub-acute exposure, most likely counteracting the parallel increase in ACE, consequently maintaining unchanged the ACE/ACE2 ratio. This may indicate a potential increased vulnerability to the entry of SARS-CoV-2 in the event of contact.

The ACE2/Ang(1-7)/MASR axis is known to counterbalance the adverse effects induced by the ACE/AngII/AT1R axis in the central nervous system. This axis exerts a protective action against ischemic and hemorrhagic strokes via increased cerebral blood flow and the attenuation of neurological deficits. Other significant effects are added, including the attenuation of endothelial function loss in cerebral arteries that occurs with aging, the increase in cerebral angiogenesis, and the attenuation of blood–brain barrier integrity alterations caused by reperfusion ischemia [59].

Moreover, as previously demonstrated [19], DEP exposure leads to an increase in COX-2, indicating that inflammation is elevated in both acute and sub-acute treatments. While the increase in HO-1 level in acute exposure may be a protective response, it is evident that DEP exposure results in a highly compromised inflammatory state in the cortex. This could lead to severe complications in the event of SARS-CoV-2 infection.

The situation in the cerebellum is the opposite of what we see in the cortex after acute DEP exposure. There is a clear decrease in both ACE2 and ACE levels.

The significant rise in ACE levels caused by sub-acute DEP treatment leads to a notable change in the ACE/ACE2 ratio, which could, in turn, result in exposure to inflammation, oxidative stress, and perhaps even severe consequences under SARS-CoV-2 infection. Indeed, the increase in the ACE level, occurring concurrently with the absence of ACE2 and HO-1 induction, indicates a potential failure in the activation of cellular defense mechanisms. The strong iNOS increase may also suggest a greater probability of severe consequences in the event of SARS-CoV-2 infection. Finally, the observed increase in s-ACE2 levels in plasma following sub-acute DEP exposure is consistent with the elevated, although not significant, AngII levels compared to sham observed in previous experiments carried out in our laboratory. 

Previous studies have shown that the increase in ACE2 shedding and the consequent increase in s-ACE2 are associated with severe myocardial dysfunction and are predictive of adverse clinical events [66]. It has also been hypothesized that s-ACE2 plays a protective role against infection by binding to the viral S glycoprotein. These observations are currently conflicting, and further study is needed to resolve them [66]. The elevated level of s-ACE2 found in seriously ill patients does not seem to protect them during viral infection [67]. The debate continues as to which is the most implicated ACE2 proteolytic shedding mechanism in SARS-CoV-2 entry into the host cell [66].

## 5. Conclusions

PM is a highly heterogeneous mixture containing particles of different sizes, shapes, and chemical compositions. Among all PM, the particles with the smallest d*a*, i.e., the UFP, are the most transportable in the atmosphere and have the greatest capacity to be inhaled through the respiratory tract.

The results of our study using an in vivo model of male BALB/c mice demonstrated that both acute and sub-acute exposures to UFP (BB or DEP) led to changes in the levels of ACE2 and/or ACE in all considered organs. 

It is possible that this may have consequences for the pathogenesis of the virus. Indeed, ACE2 acts as a protective factor for the organs, counteracting the pro-inflammatory pathways activated by ACE. However, it also acts as a receptor for SARS-CoV-2. Therefore, in the event of contact with the virus, an alteration of the ACE/ACE2 ratio in favor of ACE2 could promote its entry, while in favor of ACE, it could cause more severe symptoms under infection. 

While the exact mechanism is still unclear, evidence suggests that increased ACE2 expression may offer protection against PM cytotoxicity. However, this could potentially increase the risk of SARS-CoV-2 infection, providing a biological plausibility for epidemiological studies [30,31].

As previously observed in our research on the lungs, heart, cortex, and cerebellum [18,19], COX-2, HO-1, and iNOS levels are subject to change in all the organs under consideration. It is worth noting that these are critical proteins involved in UFP-induced inflammation and oxidative stress and that they are also closely related to the COVID-19 disease [68,69]. The results show that acute and sub-acute UFP exposures, as observed in PM2.5 sub-acute treatments [34], induce organ-specific modifications. 

Interestingly, the sub-acute exposure to PM2.5 in the lungs resulted in a significant increase in ACE2, which is not observed following treatment with BB or DEP. This underlines the greater ability of UFP to arrive in the bloodstream, spreading the damage even further away from the lungs.

The heart is particularly susceptible to DEP exposure, which consistently alters the ACE/ACE2 ratio. The liver seems more affected by acute DEP exposure, while the cerebellum is affected by sub-acute DEP exposure, as evidenced by the impairment of the ACE/ACE2 ratio. Furthermore, DEP exposure is more effective than BB in increasing COX-2, HO-1, and iNOS levels. Finally, sub-acute exposure to DEP causes an increase in s-ACE2, which might have an adverse role in the case of infection. The binding of the SARS-CoV-2 to s-ACE2 may potentially contribute to the spread of the virus to distant organs [70].

It seems plausible that UFP and SARS-CoV-2 may influence common pathways. In a “risk stratification” concept, it is possible that the chance of developing a severe form of COVID-19 may increase significantly in case of daily exposure to pollution. This could help to explain, at least in part, the virus’s greater lethality observed in highly polluted areas. Indeed, recent papers that analyze epidemiological data during the pandemic confirm this fatal correlation [50,51,52].

To gain a deeper understanding of the mechanism by which SARS-CoV2 interacts with target cells, it would be crucial to consider potential sites of interaction between the spike protein and the cell surface in addition to ACE2. Recent studies show that membrane micro-domains called lipid rafts play a critical role in facilitating viral entry. Both the receptor binding domain and the spike protein of SARS-CoV-2 are capable of binding to sialylated glycans on the mono sialic ganglioside GM1, which is enriched in the lipid rafts [71,72]. The next step will be to isolate the lipid rafts and analyze whether ACE2, following exposure to UFP, increases its colocalization with GM1. This interaction could potentially increase the effectiveness of viral entry into the cell. 

## Figures and Tables

**Table 1:** ACE/ACE2 ratios in the different organs following acute and sub-acute exposure to BB and DEP. Each ratio is expressed as fold-change to sham. Data are expressed as mean ± s.e. standard error. * *p* < 0.05 versus sham.

	Acute BB	Acute DEP	Sub-Acute BB	Sub-Acute DEP
Lungs	1.6 ± 0.38	0.9 ± 0.08	1.0 ± 0.07	1.1 ± 0.15
Heart	0.7 ± 0.12	0.6 ± 0.09 *	0.8 ± 0.11	0.7 ± 0.09 *
Liver	0.9 ± 0.14	1.6 ± 0.16 *	1.4 ± 0.17	1.0 ± 0.15
Kidneys	0.9 ± 0.17	1.2 ± 0.18	0.7 ± 0.10	0.8 ± 0.16
Cortex	1.5 ± 0.25	1.2 ± 0.18	1.0 ± 0.16	1.2 ± 0.19
Cerebellum	1.1 ± 0.09	0.7 ± 0.19	1.6 ± 0.24	2.9 ± 0.49 *

## Data Availability

The data presented in this study are available on request from the corresponding authors.

## Supplementary Materials

The following supporting information can be downloaded at https://www.mdpi.com/article/10.3390/toxics12080560/s1, Table S1: title. Chemical compositions of UFPs from different anthropogenic sources, reported in Longhin et al., 2016 [17]. Each value is expressed as mean concentrations (±SD).
