# Supplementary figures and images for: Correlation between Exposure to UFP and ACE/ACE2 Pathway: Looking for Possible Involvement in COVID-19 Pandemic
Source: Toxics. 2024 Jul 31;12(8):560. doi: 10.3390/toxics12080560 (PMC11359209; doi:10.3390/toxics12080560)

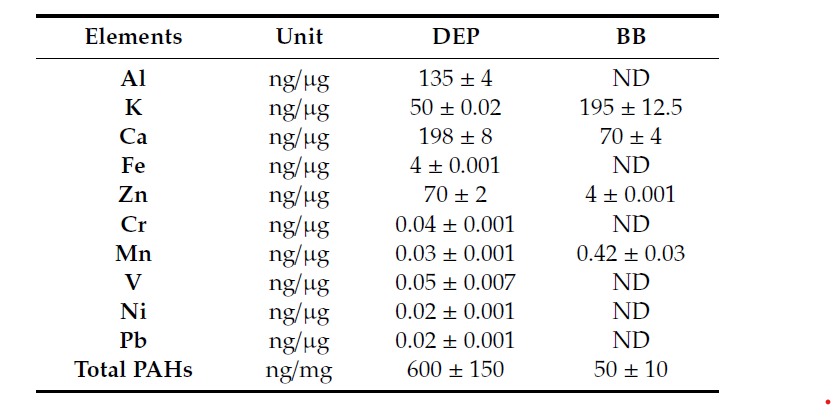

Supplement: Supplementary file 1 [file toxics-12-00560-s001.zip › toxics-3021828-supplementary.png]
